# The scientific knowledge of three large language models in cardiology: multiple-choice questions examination-based performance

**DOI:** 10.1097/MS9.0000000000002120

**Published:** 2024-05-06

**Authors:** Ibraheem Altamimi, Abdullah Alhumimidi, Salem Alshehri, Abdullah Alrumayan, Thamir Al-khlaiwi, Sultan A. Meo, Mohamad-Hani Temsah

**Affiliations:** a College of Medicine; b Department of Physiology; c Evidence-Based Health Care and Knowledge Translation Research Chair, Family and Community Medicine Department, College of Medicine, King Saud University; d Pediatric Intensive Care Unit, Pediatric Department, College of Medicine, King Saud University Medical City; e College of Medicine, King Saud Bin Abdulaziz University for Health and Sciences, Riyadh, Saudi Arabia

**Keywords:** AI assessment, artificial intelligence, cardiology, MCQ assessment, medical education

## Abstract

**Background:**

The integration of artificial intelligence (AI) chatbots like Google’s Bard, OpenAI’s ChatGPT, and Microsoft’s Bing Chatbot into academic and professional domains, including cardiology, has been rapidly evolving. Their application in educational and research frameworks, however, raises questions about their efficacy, particularly in specialized fields like cardiology. This study aims to evaluate the knowledge depth and accuracy of these AI chatbots in cardiology using a multiple-choice question (MCQ) format.

**Methods:**

The study was conducted as an exploratory, cross-sectional study in November 2023 on a bank of 100 MCQs covering various cardiology topics that was created from authoritative textbooks and question banks. These MCQs were then used to assess the knowledge level of Google’s Bard, Microsoft Bing, and ChatGPT 4.0. Each question was entered manually into the chatbots, ensuring no memory retention bias.

**Results:**

The study found that ChatGPT 4.0 demonstrated the highest knowledge score in cardiology, with 87% accuracy, followed by Bing at 60% and Bard at 46%. The performance varied across different cardiology subtopics, with ChatGPT consistently outperforming the others. Notably, the study revealed significant differences in the proficiency of these chatbots in specific cardiology domains.

**Conclusion:**

This study highlights a spectrum of efficacy among AI chatbots in disseminating cardiology knowledge. ChatGPT 4.0 emerged as a potential auxiliary educational resource in cardiology, surpassing traditional learning methods in some aspects. However, the variability in performance among these AI systems underscores the need for cautious evaluation and continuous improvement, especially for chatbots like Bard, to ensure reliability and accuracy in medical knowledge dissemination.

## Introduction

Highlights

- ChatGPT 4.0 displayed greater knowledge of cardiology with a highest score 88%, outperforming Bing and Bard with score 61%, and 46%, respectively.
- ChatGPT 4.0 outperformed Bing and Bard knowledge of coronary heart disease and arrhythmias.
- ChatGPT 4.0 outshined in heart failure and cardiovascular drugs with 92.8 and 85.7% scores, respectively, while Bing and Bard showed significantly lower intellect.
- Despite diverse performances, all three ChatGPT 4.0, Bing and Bard chatbots showed significant contributions to various areas of the field studied but displayed deficiency in comprehensive cardiology knowledge.

The artificial intelligence (AI) chatbot is a computer-based program designed to simulate conversation with human users, typically through text-based interfaces. These chatbots use natural language processing and machine learning algorithms to understand user inputs, interpret meaning, and generate appropriate responses. Google’s Bard, OpenAI’s ChatGPT, and Microsoft’s Bing Chatbot are leading the charge in the domain of AI chatbots, each renowned for their ability to effectively generate and disseminate information. Google’s Bard, leveraging the power of LaMDA, and ChatGPT, a generative pretrained transformer chatbot, along with Microsoft’s Bing Chatbot, are all fueled by vast datasets enabling them to produce nuanced and realistic responses^1,2^. As advanced AI tools, they excel in swiftly collecting, interpreting, and presenting data across a diverse range of topics. Their increasing use in drafting articles, conducting research, and solving complex queries marks their significance in academic and professional spheres^3–5^.

In the field of cardiology, within academia and research, the impact of these AI chatbots, Google’s Bard, OpenAI’s ChatGPT, and Microsoft’s Bing Chatbot has not been investigated for evaluation the new opportunities for students, educators, and industry professionals^6^. However, their integration into traditional educational and research frameworks also poses challenges, particularly concerning the potential erosion of critical thinking skills^7,8^. There is a distinct lack of in-depth literature evaluating their efficacy specifically within cardiology^9^. The debate over the use of these chatbots in cardiology research and education is vibrant, with diverse viewpoints on their suitability and best use cases^10^.

Recent research has started to shed light on the role of these chatbots in both basic and clinical medical sciences, particularly in cardiology and related technologies but still more is needed in this field. The capabilities and potential applications of Google’s Bard, OpenAI’s ChatGPT, and Microsoft’s Bing Chatbot in medical sciences, especially cardiology, are topics of ongoing discussion and exploration. However, literature is lacking to investigate the knowledge level of these AI tools in cardiology. This study aims to evaluate the knowledge of these AI chatbots in the field of cardiology through a multiple-choice question (MCQ) examination format, to gain insights into their strengths and limitations in this specialized area.

## Methods

### Study design and setting

This cross-sectional, analytical study was conducted in the College of Medicine, King Saud University during November 2023.

### Establishing MCQ bank

The creation of the MCQ bank was a meticulous process undertaken by the research team. The team sourced questions from a variety of authoritative textbooks and question banks. These sources included the renowned ‘Guyton and Hall Textbook of Medical Physiology’, ‘First Aid for the USMLE Step 1’, ‘First Aid for the USMLE Step 2’, ‘AMBOSS Step 1’, ‘UWorld Step 1’, ‘UWorld Step 2’, as well as various university examination archives. From these comprehensive and respected resources, the team successfully compiled a total of 800 MCQs.

Each MCQ, along with its corresponding answer key, underwent a thorough review to ensure relevance and accuracy in relation to the subject matter. The questions were designed to be scenario-based, comprising four answer choices each, with only one correct answer. Where necessary, the scenarios of the questions were adapted, taking care not to alter their original meaning or the correctness of the key answers. This approach ensured the creation of a comprehensive and reliable MCQ bank, tailored for evaluating the knowledge in the specified field^11^.

### Selection of MCQs examination

**Figure 1:**
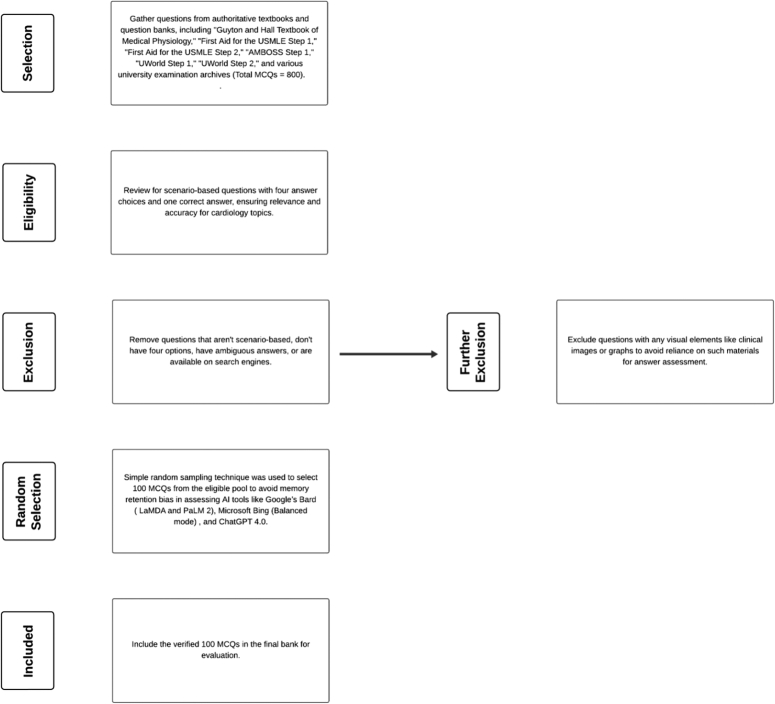
Flow diagram of the selection of multiple-choice questions.

In this study, a total of 100 MCQs from various cardiology topics were selected for evaluation from a pool of 800 questions sourced from various question banks (Fig. 1), utilizing a simple random sampling technique to ensure a representative selection of questions across the discipline. The MCQs were based on the physiology, pathophysiology of cardiac diseases, and the diagnosis and management of various cardiac disorders. The following subtopics were included arrhythmia, coronary heart disease (CHD), valvular heart disease, heart failure, cardiovascular drugs, myopericardial diseases, hypertension, and congenital heart disease. We assessed the knowledge level of Google’s Bard, Microsoft Bing, and ChatGPT 4.0. The questions were entered manually one by one, and a fresh Chatbot session was started for each entry to avoid memory retention bias. Globally, universities and examination boards often employ MCQs in various assessments. These questions are a staple in medical education, serving as effective tools for facilitating learning. Medical schools and licensing authorities across the globe use MCQ-based examinations to assess the knowledge as a standard practice^12^.

The role of MCQs in accurately gauging cognitive abilities, critical thinking, and problem-solving skills is crucial. They offer a comprehensive means of assessing critical thinking, effectively evaluating higher cognitive functions. MCQs are instrumental in testing a candidate’s capacity to correlate different concepts and scrutinize evidence in diverse contexts. Furthermore, they establish a solid structure for measuring advanced cognitive functions by enhancing critical thinking and the practical application of knowledge^13,14^.

### Ensuring uniqueness and relevance of MCQs

The MCQs underwent a thorough review process to verify that their answers, associated content, or explanations were not accessible via search engines. Additionally, any questions in the test that incorporated visual elements, including clinical images, graphs, or illustrations, were excluded from the study.

### Data collection

Data collection took place over a period of 1 week, from 10 November to 18 November 2023. Each MCQ was input individually into Google’s Bard, Microsoft Bing (Balanced mode), and ChatGPT 4.0 by two members of the research team, and the respective responses from each were meticulously documented. The first response given by each tool was considered final, and the feature to ‘regenerate response’ was not utilized. Scoring of the responses was binary: a score of ‘0’ indicated an incorrect answer, while a score of ‘1’ signified a correct response, all in accordance with a previously established answer key.

### Ethical considerations

This study, which compiled MCQs from various banks and textbooks and did not involve human or animal subjects, thereby negating the need for ethical clearance, is reported in line with the STROCSS criteria^15^.

### Statistical analysis

Categorical variables were characterized using frequency distributions and percentages. Associations between categorical variables were examined using the *χ*^2^ test of independence. Data analyses were performed using IBM SPSS Statistics software, version 28. A *P*-value of less than 0.05 was considered statistically significant.

## Results

**Figure 2:**
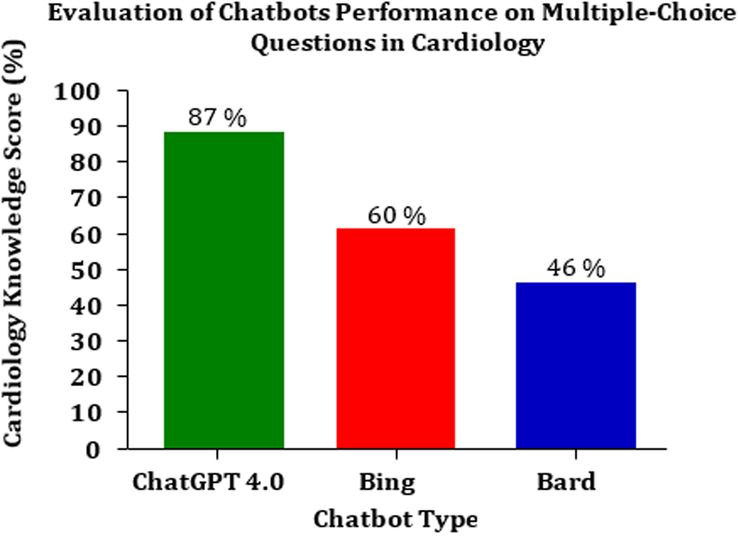
Evaluation of chatbots performance on multiple-choice questions in cardiology.

The bar chart shown in Figure 2 presents a comparative analysis of the performance of various chatbots on cardiology MCQs. From the data, ChatGPT 4.0 exhibits the highest knowledge score with 87%, indicating a superior grasp of cardiology topics among the tested chatbots. The Bing chatbot follows with a knowledge score of 60%, demonstrating a moderate level of understanding. Lastly, the Bard chatbot scored 46%, which suggests a relatively lower proficiency in cardiology compared to its counterparts. The results reflect the varying capabilities of these chatbots in processing and accurately responding to specialized knowledge queries within the field of cardiology. This variation in performance may be due to differences in the underlying algorithms, knowledge databases, and learning capabilities of the respective chatbots.

Table 1 presents a detailed descriptive analysis of the performance of three chatbots—ChatGPT, Bing, and Bard—on a cardiology knowledge assessment comprising MCQs. The analysis reveals that ChatGPT scored the highest across the board, achieving an 87% success rate on the overall cardiology knowledge test, thereby surpassing the threshold of 50% that constitutes a passing score. Bing and Bard followed with scores of 60 and 46%, respectively, with Bard falling below the passing mark, thus failing to answer at least half of the posed questions satisfactorily.

**Table 1:** The number of correctly answered questions by chatbots for different cardiology aspects.

	Correctly answered questions *n*=193		
All test questions, *n*=100 questions	ChatGPT4	Bing	Bard
Overall knowledge test	87/100 (87%)	60/100 (60%)	46/100 (46%)
Arrhythmia	16/19 (84.2%)	13/19 (68%)	10/19 (52%)
Coronary heart disease	23/27 (85.2%)	14/27 (51.9)	11/27 (40.7%)
Ventricular heart disease	15/17 (88.2%)	10/17 (85.8%)	9/17 (52.9%)
Heart failure	13/14 (92.8%)	11/14 (78.6%)	7/14 (50%)
Cardiovascular drugs	6/7 (85.75)	2/7 (28.6%)	1/7 (14.3%)
Myopericardial diseases	6/6 (100%)	6/6 (100%)	4/6 (66.7%)
Hypertension	8/10 (80%)	4/10 (40%)	4/10 (40%)

Focusing on the domain of arrhythmias, ChatGPT maintained its lead with a score of 85.2%, whereas Bing and Bard lagged with scores of 51.9 and 40.7%, respectively. Similar trends were observed in the CHD knowledge domain, where ChatGPT again outperformed with an 85.2% score, Bing secured 51.9%, and Bard scored 40.7%, once more below the passing threshold.

In the evaluation of ventricular heart disease knowledge, ChatGPT and Bing exhibited strong performances with scores of 88 and 85.8%, respectively, while Bard showed a lower proportion of correct answers 52.9%. When assessing knowledge of heart failure, ChatGPT achieved the highest mark at 92.8%, followed by Bing at 78.6%, and Bard at the baseline passing score of 50%.

Regarding the understanding of cardiovascular drugs, ChatGPT4.0 demonstrated the highest competency with an 85.7% score. However, Bing and Bard showed significantly lower proficiency with scores of 28.6 and 14.3%, respectively. Both ChatGPT and Bing displayed full mastery in answering questions related to myocardial disease, each scoring 100%, whereas Bard showed partial knowledge with a 66.7% score.

Lastly, in the hypertension knowledge category, ChatGPT4.0 scored 80%, while Bing and Bard each scored 40%, indicating a failure to answer a majority of the hypertension-related questions correctly.

The results, as illustrated in Table 2, indicate that ChatGPT 4.0 significantly outperformed Bing and Bard in the overall cardiology knowledge assessment, with a *P*-value less than 0.001, denoting a high level of statistical significance. In contrast, a *χ*^2^ test for independence revealed no significant difference among the chatbots regarding their knowledge of cardiac arrhythmias, as indicated by a *P*-value of 0.112.

**Table 2:** Bivariate analysis of different chatbots on their cardiology knowledge aspects.

	Incorrect answers, *n*=107	Correct answers, *n*=193	Test statistic	*P*
All test questions, *n*=100 questions				
ChatGPT	13 (12.1)	87 (45.1)	*χ*^2^(2)=37.86	<0.001
Bing	40 (37.4)	60 (31.1)		
Bard	54 (50.5)	46 (23.8)		
Arrhythmia knowledge test, *n*=19 questions				
ChatGPT	3 (16.7)	16 (41)	χ^2^(2)=4.39	0.112
Bing	6 (33.3)	13 (33.3)		
Bard	9 (50)	10 (25.6)		
Coronary heart disease knowledge, *n*=27 questions				
ChatGPT	4 (12.1)	23 (47.9)	χ^2^(2)=11.97	0.003
Bing	13 (39.4)	14 (29.2)		
Bard	16 (48.5)	11 (22.9)		
VHD knowledge test, *n*=17 questions				
ChatGPT	2 (11.8)	15 (44.1)	χ^2^(2)=5.050	0.065
Bing	7 (41.2)	10 (29.4)		
Bard	8 (47.1)	9 (26.5)		
Knowledge of heart failure, *n*=14 questions				
ChatGPT	1 (9.1)	13 (41.9)	χ^2^(2)=6.89	0.032
Bing	3 (27.3)	11 (35.5)		
Bard	7 (63.6)	7 (22.6)		
Knowledge of cardiovascular drugs, *n*=7 questions				
ChatGPT	1 (8.3)	6 (66.7)	χ^2^(2)=8.17	0.017
Bing	5 (41.7)	2 (22.2)		
Bard	6 (50)	1 (11.1)		
Knowledge of myopericardial diseases, *n*=6 questions				
ChatGPT	0	6 (37.5)	χ^2^(2)=8.17	0.085
Bing	0	6 (37.5)		
Bard	2 (100)	4 (25)		
Knowledge of hypertension disease, *n*=10 questions				
ChatGPT	2 (14.3)	8 (50)	χ^2^(2)=4.53	0.104
Bing	6 (42.9)	4 (25)		
Bard	6 (42.9)	4 (25)		

Furthermore, ChatGPT 4.0 demonstrated significantly greater expertise in CHD compared to Bing and Bard, with a *P*-value of 0.003, suggesting a substantial difference in proficiency within this specific domain.

The analysis did not find a significant difference in the knowledge of valvular heart disease among the chatbots, with a *P*-value of 0.065, which is above the conventional threshold for statistical significance.

When examining knowledge in the area of heart failure, Bard was statistically more likely to answer incorrectly when compared to ChatGPT4.0 and Bing, as shown by a *P*-value of 0.032. This suggests a notable disparity in the quality of responses between the chatbots for this subject area. Additionally, ChatGPT4.0 was significantly more adept at answering questions related to cardiovascular drugs than Bing and Bard, supported by a *P*-value of 0.017. However, the analysis indicated no significant differences between the chatbots in their knowledge of myopericardial diseases and hypertension, with *P*-values exceeding 0.050 for each category, implying a comparable level of understanding among the chatbots for these conditions.

## Discussion

Google’s Bard, Microsoft Bing, and ChatGPT have garnered significant interest from various sectors, including the general public, students, academicians, researchers, and the broader scientific community. These advanced language models are known for their ability to quickly and effectively respond to queries across numerous disciplines. They have become invaluable tools in enhancing scientific understanding, crafting essays, and providing detailed explanations^16^. There is ongoing discussion regarding the levels of knowledge and intelligence possessed by Google’s Bard, Microsoft Bing, and ChatGPT. However, there is a noticeable gap in the literature when it comes to evaluating and comparing their knowledge specifically in the area of Cardiology.

To the best of our understanding, this research represents a pioneering effort in directly comparing the knowledge capabilities of three distinct AI tools—ChatGPT, Bing, and Bard—specifically in the domain of Cardiology. This comparative analysis is unique in its approach, focusing on factual knowledge within this medical field. Conducting this evaluation concurrently across the three AI platforms enhances the validity and reliability of both the examination content and the capabilities of the AI tools under study. This methodology offers a novel perspective in assessing AI proficiency in medical knowledge, particularly in cardiology.

A study by Meo *et al*.^17^ revealed that ChatGPT scored 74% in basic medical sciences and 70% in clinical medical sciences, with an overall score of 72% across both areas. Duong and Solomon^16^ reported a score of 68.2% for ChatGPT, while Gilson *et al*.^18^ observed varying scores of 44%, 42%, 64.4%, and 57.8% in different examinations.

In this study, focusing on cardiology, ChatGPT4.0 scored 87%, with Bing significantly behind at 60% and Bard scored the least with 46%. Beaulieu-Jones *et al*.^19^ found ChatGPT-4’s accuracy to be 71 and 68% in MCQs. Mihalache *et al*.^20^ noted that ChatGPT answered 46% of questions correctly, and Antaki *et al*.^21^ reported scores of 55.8% in basic and clinical science and 42.7% in ophthalmology. Friederichs *et al*.^22^ found ChatGPT scored 65.5% in their examination. In the discussion of the findings by Meo *et al*.^11^, it was noted that when evaluating the comprehension of endocrinology and diabetes technology, ChatGPT achieved a score of 52 out of 10. In close comparison, Bard received a score of 49 out of 100.

In the light of these findings, particularly considering the benchmarks set by significant international exams, the performance of ChatGPT4.0, Bing, and Bard in the field of cardiology is noteworthy. ChatGPT4.0 achieved an impressive score of 87%, suggesting that it demonstrates a level of proficiency that may potentially meet or even exceed the passing criteria for international examinations such as the USMLE^23^ and MCCQE^24^, where the passing mark ranges from 60 to 70%. Bing, with a score of 60%, is on the cusp of this threshold, almost meeting the standard required to pass these exams. In contrast, Bard, scoring 46%, falls short of the passing mark, indicating a failure to meet the standards set by these international examinations in cardiology.

The advancement of AI technology heralds exciting opportunities for exploration, laying a foundational platform for comprehending the medical logic underlying various conditions. There is a growing need for more in-depth research to assess and understand the abilities of Google’s Bard, Microsoft Bing, and ChatGPT in addressing complex medical reasoning questions. As this technology evolves, it could lead to the development of innovative educational methods in medicine, leveraging the full potential of these AI tools across different fields, such as cardiology.

Recently, advancements in AI have enabled chatbots like ChatGPT to not only process text but also to interpret auditory inputs, visualize data, and engage in spoken dialogue. This enhancement in capabilities, such as the ability to listen to and analyze heart sounds, could significantly enrich the way these chatbots contribute to the assessment and learning of medical knowledge. While AI models excel at processing vast information, their reasoning in complex medical scenarios is not failproof, and a correct guess could be mistakenly seen as accurate diagnosis or understanding, leading to potential misinterpretations.

### Study strengths and limitations

The robustness of this investigation into the capabilities of Google’s Bard, Microsoft Bing, and ChatGPT 4.0 lies in its pioneering nature; it is the first study to conduct a comparative analysis of three distinct chatbots in the realm of cardiology knowledge. The ability of these chatbots to exhibit knowledge and respond to queries related to cardiology is particularly critical. Bard and ChatGPT have the potential to evolve into accessible tools that can deliver vital information, addressing a pressing need for immediate, accurate medical knowledge dissemination.

Nevertheless, there are few limitations to this study. The scope of the assessment was confined to a limited selection of MCQs, which may not accurately reflect the nuanced understanding required in real-world clinical education settings. Additionally, the MCQs utilized were text-only scenarios that did not incorporate diagnostic images, which are integral to comprehensive cardiology education. This omission could affect the applicability of the chatbots’ performance to actual clinical practice, where visual diagnostic skills are essential.

## Conclusions

The study reveals that while chatbots hold promise as tools for disseminating cardiology knowledge, there is a spectrum of efficacy among them. ChatGPT 4.0, with its superior performance, could potentially serve as an auxiliary educational resource in cardiology, complementing traditional learning methods. However, the variability in chatbot performances indicates that reliance on these AI systems should be tempered with cautious evaluation and that there is substantial room for improvement, especially for chatbots like Bard, to ensure reliability and accuracy in medical knowledge dissemination.

## Ethical approval

Since the research did not involve human or animal subjects, there was no necessity for ethical clearance.

## Consent

Since the research did not involve human or animal subjects, there was no necessity for ethical clearance.

## Sources of funding

The authors have not declared a specific grant for this research from any funding agency in the public, commercial or not-for-profit sectors.

## Author contribution

I.A. and S.M.: conceived the research idea and contributed to the data acquisition; I.A., M.T., A.A., A.A., and S.A.: contributed to the study design; I.A.: conducted the analyses and drafted the manuscript; T.A.: is responsible for the overall content as guarantor. All the authors contributed to the interpretation of the work and made critical revision of the manuscript for key intellectual content. All the authors have read and approved the submitted version of the manuscript.

## Conflicts of interest disclosure

The authors report no personal or financial conflict of interests to declare.

## Research registration unique identifying number (UIN)

Since the research did not involve human or animal subjects, there was no necessity for ethical clearance.

## Guarantor

Ibraheem Altamimi.

## Data availability statement

Data will be made available upon reasonable request directed to corresponding author.

## Provenance and peer review

None.

## Presentation

None.

## References

[1] Rahaman MS Ahsan MMT Anjum N . The AI race is on! Google’s Bard and OpenAI’s ChatGPT head to head: an opinion article. Mizanur and Rahman, Md Nafizur, The AI Race is on 2023:3–5.

[2] Salvagno M Taccone FS Gerli AG . Can artificial intelligence help for scientific writing? Crit Care 2023;27:1–5. 36841840 10.1186/s13054-023-04380-2 PMC9960412

[3] Hutson M . Could AI help you to write your next paper? Nature 2022;611:192–193. 36316468 10.1038/d41586-022-03479-w

[4] Ram B Pratima Verma PV . Artificial intelligence AI-based Chatbot study of ChatGPT, Google AI Bard and Baidu AI. World J Adv Engineer Technol Sci 2023;8:258–261.

[5] Altamimi I Altamimi A Alhumimidi AS . Snakebite advice and counseling from artificial intelligence: an acute venomous snakebite consultation with ChatGPT. Cureus 2023;15:6. 10.7759/cureus.40351 PMC10339276 37456381

[6] Nakaya Y Higaki A Yamaguchi O . ChatGPT’s ability to classify virtual reality studies in cardiology. Eur Heart J Digit Health 2023;4:141–142; ztad026. 37265861 10.1093/ehjdh/ztad026 PMC10232268

[7] Aydin Ö . Google Bard generated literature review: metaverse. J AI 2023;7:1–14.

[8] Rahman MM Watanobe Y . ChatGPT for education and research: Opportunities, threats, and strategies. Appl Sci 2023;13:5783.

[9] Skalidis I Cagnina A Luangphiphat W . ChatGPT takes on the European Exam in Core Cardiology: an artificial intelligence success story? Eur Heart J Digit Health 2023;4:279–281. 37265864 10.1093/ehjdh/ztad029 PMC10232281

[10] Fernández-Cisnal A Lopez-Ayala P Miñana G . Performance of an artificial intelligence chatbot with web search capability in cardiology-related assistance: a simulation study. Revista espanola de cardiologia (English ed) 2023;76:1065–1067. 37506970 10.1016/j.rec.2023.06.008

[11] Meo SA Al-Khlaiwi T AbuKhalaf AA . The scientific knowledge of Bard and ChatGPT in endocrinology, diabetes, and diabetes technology: multiple-choice questions examination-based performance. J Diabetes Sci Technol 2023:2–3; 19322968231203987. 10.1177/19322968231203987 PMC12035228 37798960

[12] Palmer EJ Devitt PG . Assessment of higher order cognitive skills in undergraduate education: modified essay or multiple choice questions? Research paper. BMC Med Educ 2007;7:1–7. 18045500 10.1186/1472-6920-7-49 PMC2148038

[13] Liu Q Wald N Daskon C . Multiple-choice questions (MCQs) for higher-order cognition: Perspectives of university teachers. Innov Educat Teaching Int 2023:1–13. doi:10.1080/14703297.2023.2222715

[14] Bhayana R Krishna S Bleakney RR . Performance of ChatGPT on a radiology board-style examination: insights into current strengths and limitations. Radiology 2023;307:230582. 10.1148/radiol.230582 37191485

[15] Mathew G Agha R Albrecht J . STROCSS 2021: strengthening the reporting of cohort, cross-sectional and case-control studies in surgery. Int J Surg Open 2021;37:100430. 10.1016/j.ijsu.2021.106165 34774726

[16] Duong D Solomon BD . Analysis of large-language model versus human performance for genetics questions. Eur J Hum Genet 2023;32:1–3. 10.1038/s41431-023-01396-8 PMC10999420 37246194

[17] Meo SA Al-Masri AA Alotaibi M . ChatGPT knowledge evaluation in basic and clinical medical sciences: multiple choice question examination-based performance. Healthcare, MDPI 2023;11:2046. 10.3390/healthcare11142046 PMC10379728 37510487

[18] Gilson A Safranek CW Huang T . How does ChatGPT perform on the United States medical licensing examination? The implications of large language models for medical education and knowledge assessment. JMIR Med Educ 2023;9:e45312. 36753318 10.2196/45312 PMC9947764

[19] Beaulieu-Jones BR Shah S Berrigan MT . Evaluating capabilities of large language models: performance of GPT4 on surgical knowledge assessments. medRxiv 2023:10–11. 10.1016/j.surg.2023.12.014 PMC10947829 38246839

[20] Mihalache A Popovic MM Muni RH . Performance of an artificial intelligence chatbot in ophthalmic knowledge assessment. JAMA Ophthalmol 2023;141:589. 37103928 10.1001/jamaophthalmol.2023.1144 PMC10141269

[21] Antaki F Touma S Milad D . Evaluating the performance of chatgpt in ophthalmology: An analysis of its successes and shortcomings. Ophthalmol Sci 2023;3:100324. 37334036 10.1016/j.xops.2023.100324 PMC10272508

[22] Friederichs H Friederichs WJ März M . ChatGPT in medical school: how successful is AI in progress testing? Med Educ Online 2023;28:2220920. 37307503 10.1080/10872981.2023.2220920 PMC10262795

[23] USMLE , Scoring & score reporting. 2023. Accessed 27 July 2023. https://www.usmle.org/bulletin-information/scoring-and-score-reporting

[24] MCCQE , Outline of MCCQE Part 1 Exam. 2023. Accessed 27 July 2023. https://www.aceqbank.com/mccqe-part-1-exam-outline-2021/

